# Implementation of WirelessHART in the NS-2 Simulator and Validation of Its Correctness

**DOI:** 10.3390/s140508633

**Published:** 2014-05-16

**Authors:** Pouria Zand, Emi Mathews, Paul Havinga, Spase Stojanovski, Emiliano Sisinni, Paolo Ferrari

**Affiliations:** 1 Pervasive Systems Group, Faculty of Electrical Engineering, Mathematics and Computer Science, University of Twente, P.O. Box 217, Enschede 7500AE, The Netherlands; E-Mails: e.mathews@utwente.nl (E.M.); p.j.m.havinga@utwente.nl (P.H.); 2 Deloitte Management Consulting, Laan van Kronenburg 2, Amstelveen 1183AS, The Netherlands; E-Mail: sstojanovski@deloitte.nl; 3 Department of Electronics for the Automation, University of Brescia, Via Branze 38, 25123 Brescia, Italy; E-Mails: emiliano.sisinni@ing.unibs.it (E.S.); paolo.ferrari@ing.unibs.it (P.F.)

**Keywords:** WirelessHART (Highway Addressable Remote Transducer), NS-2 (network simulator 2), realistic simulation, validation, IEEE 802.15.4e

## Abstract

One of the first standards in the wireless sensor networks domain, WirelessHART (HART (Highway Addressable Remote Transducer)), was introduced to address industrial process automation and control requirements. This standard can be used as a reference point to evaluate other wireless protocols in the domain of industrial monitoring and control. This makes it worthwhile to set up a reliable WirelessHART simulator in order to achieve that reference point in a relatively easy manner. Moreover, it offers an alternative to expensive testbeds for testing and evaluating the performance of WirelessHART. This paper explains our implementation of WirelessHART in the NS-2 network simulator. According to our knowledge, this is the first implementation that supports the WirelessHART network manager, as well as the whole stack (all OSI (Open Systems Interconnection model) layers) of the WirelessHART standard. It also explains our effort to validate the correctness of our implementation, namely through the validation of the implementation of the WirelessHART stack protocol and of the network manager. We use sniffed traffic from a real WirelessHART testbed installed in the Idrolab plant for these validations. This confirms the validity of our simulator. Empirical analysis shows that the simulated results are nearly comparable to the results obtained from real networks. We also demonstrate the versatility and usability of our implementation by providing some further evaluation results in diverse scenarios. For example, we evaluate the performance of the WirelessHART network by applying incremental interference in a multi-hop network.

## 1. Introduction

Despite the advancement of the realm of wireless sensor networks, their adoption by the industry for factory automation and process control applications remained limited. This all changed, when in 2007, the HART (Highway Addressable Remote Transducer) Communication Foundation [1] developed WirelessHART, the first open, international standard to fulfill industrial requirements. Using a self-organizing and self-healing mesh network architecture, it establishes a secure and reliable wireless communication protocol. It is backward compatible with the widely-used wired HART (Highway Addressable Remote Transducer) protocol: the global standard for sending and receiving digital information over analogue wires between monitoring and control systems. The WirelessHART standard has gained the confidence of the industry, and it has been increasingly adopted over the last few years [2].

The International Society of Automation (ISA) considers six classes of applications, from critical control to monitoring, in which the importance of the message timeliness and quality of service (QoS) requirements decreases from Class 0 to 5 in Table 1 [3]. WirelessHART supports industrial applications ranging from Class 2 to 5 [3].

Being the first open standard, WirelessHART can be used as a reference point to evaluate other wireless protocols in the industrial domain. This can be conveniently achieved by implementing the WirelessHART protocol in a network simulator. In addition, such implementations serve as a basis for further extensions and improvements of the protocol itself. Furthermore, to test and analyze the protocol easily, simulation provides a good alternative to expensive testbeds that need to be setup in real industrial environments. These factors motive us to work on implementing the WirelessHART simulator protocol. To that end, we choose one of the most popular network simulators, NS-2 [4], for our implementation.

Although WirelessHART has been partially implemented in other simulators [5], to the extent of our knowledge, this is the first and complete WirelessHART simulator. This means that in our simulator, we implement the entire WirelessHART stack (all OSI (Open Systems Interconnection model) layers) of field devices and access points and also the algorithms for centralized network management. A preliminary version of the simulator has been discussed in [6]. In this paper, we present the implementation of the WirelessHART simulator, which adds a security layer to provide secure and reliable communications. In addition, we validate the simulator by using sniffed/captured traffic from a real WirelessHART network.

The rest of the article is organized as follows: Section 2 provides background information on the concepts used in WirelessHART and summarizes related works. Section 3 explains the WirelessHART architecture, while Section 4 provides the implementation details of the WirelessHART device stack and the WirelessHART central network management algorithm. Methods on validating the simulator are discussed in Section 5. Experimental analysis of the real and simulated networks demonstrating the similarities and differences of network management algorithms is given in Section 6. Additional experiments in a multi-hop simulated scenario demonstrating the usability of the simulator are described in Section 7. Section 8 describes how the simulation tools can be used, and finally, Section 9 concludes the article.

## 2. Background and Related Work

In any industrial network, the major concern is to provide real-time and reliable communications. Resource reservation is one of the techniques that can facilitate real-time communication. Channel hopping and multipath routing are two suitable schemes to provide reliable communication by mitigating the deep fading and external interference. These schemes were first proposed in the time synchronized mesh protocol (TSMP) [7] and were later adopted in the WirelessHART standard. In this section, we provide some background information on TSMP and provide a summary of relevant works on WirlessHART simulation.

### 2.1. Time Synchronized Mesh Protocol (TSMP)

TSMP is the first medium access and networking protocol designed for low-power/low-bandwidth reliable communication that utilizes all of the above-mentioned techniques. TSMP concepts are used in several existing industrial wireless technologies, such as WirelessHART [1], ISA100.11a [8] and IEEE 802.15.4e (Time Slotted Channel Hopping (TSCH) mode) [9]. IEEE 802.15.4e TSCH mode is a MAC (Media Access Controll) amendment of the 802.15.4-2006 standard [10] to support industrial applications. TSCH is based on a time-slotted mechanism, where a schedule dictates on what slot and which channel a node should transmit/receive data to/from a particular neighbor.

**Figure 1.**
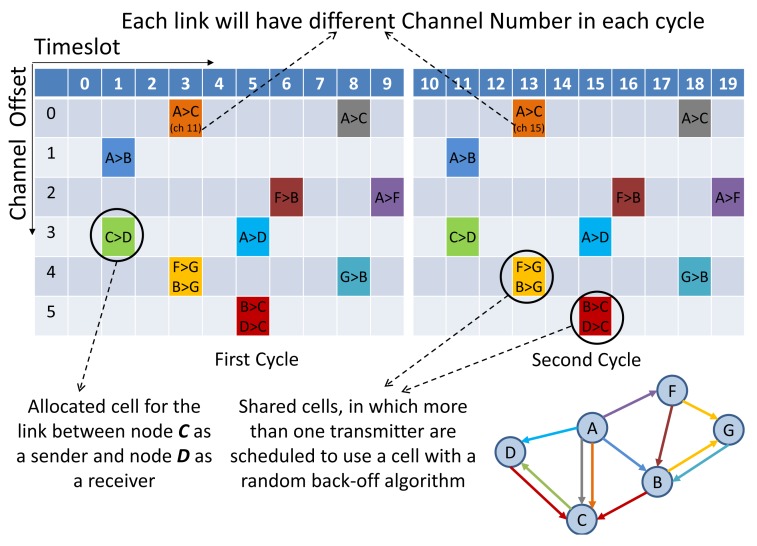
Time Slotted Channel Hopping (TSCH) slot-channel matrix for a sample network.

TSMP divides the wireless channel into time and frequency. Time is divided into superframes, which consist of a collection of discrete time slots. Figure 1 illustrates the TSMP matrix for a sample network with a superframe of 10 slots. A single element in the TSMP superframe is called a cell. A link is a transaction that occurs within a cell. Link information consists of a superframe ID (Identification), source and destination IDs, a slot number referring to the beginning of the superframe and a channel offset. The two nodes at either end of the link communicate periodically once in every superframe. If only one transmitter is scheduled, the cell is contention-free. If multiple transmitters are scheduled for transmitting to the same device in a shared cell simultaneously, a random back-off algorithm can be used. Multiple links can be allocated from one node to another in different cells. For example, two Tx-links from Node A to Node C are shown in Figure 1. TSMP links hops pseudo-randomly over a set of predefined channels. The radio channel used for communication is determined by considering the timeslot number (ASN), channel offset and channel hopping sequence, which can be formulated as follows:

(1) ActualCh#=Channel Hopping Sequence((ASN+Channel Off set)%Number of Channels)

**Figure 2.**
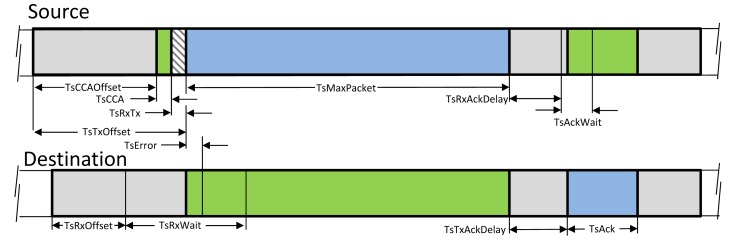
Timing of a dedicated Time Synchronized Mesh Protocol (TSMP) timeslot [1].

Figure 2 depicts the specific timing requirement inside a TSMP timeslot. The scheduled communication in a timeslot between two nodes relies on accurate time synchronization across the network. The network devices should have the same notion of when each timeslot begins and ends. TSMP, unlike the IEEE 802.15.4, which uses the beacon-based synchronization scheme, relies on exchanging the timing offset information of the received and sent packets to provide synchronization. The mechanisms for time synchronization are described in [1].

**Figure 3.**
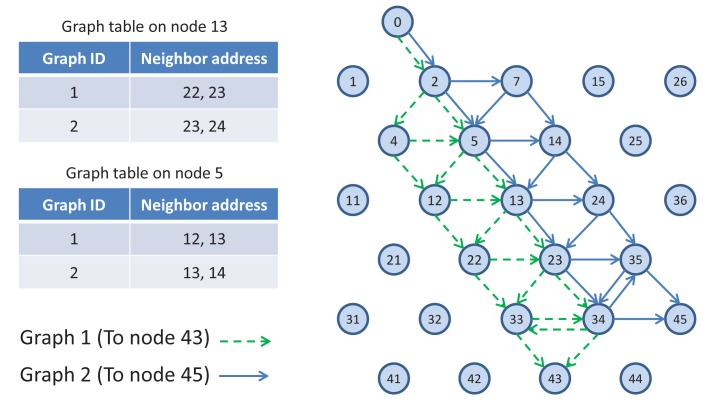
Graph routing sample.

TSMP works on graph routing-based schemes. A graph is a routing structure that establishes directed end-to-end connection among devices. Each destination has its own graph, and several sources can share the same graph. Each graph in a network is identified with a unique graph ID. Figure 3 illustrates the graph routing. In this figure, Node 0 uses graphs with IDs 1 and 2 to communicate with Nodes 43 and 45, respectively. When a source node wants to send a packet to a destination, the graph ID will be included in the packet header to enable routing to the destination. At any node in the path, multiple next hops could be specified in a mesh graph; path diversity is directly built-in [7]. In Figure 3, for example, an intermediate Node 5 may forward a packet identified with Graph ID 1 to Node 12 or Node 13 and may forward a packet identified with Graph ID 2 to Node 13 or Node 14.

### 2.2. Related Work

Existing implementations of WirelessHART are partial. Nobre *et al.* [5] have developed a WirelessHART module for the NS-3 (Network Simulator 3) simulator. The focus of that work was on implementing the physical layer of WirelessHART to use it as a basis for developing other layers, such as the MAC and application layer. In [11], the authors report on the development of the physical and MAC layer of WirelessHART in OMNET++ [12]. This tool analyzes the effect of interference on the WirelessHART network. However, they did not implement the full WirelessHART stack nor the network management algorithms. In [13], the authors did implement a WirelessHART simulator based on TrueTime, an open source MATLAB/Simulink-based tool for simulating networks, to study the clock drift in process control. However, in that research, the WirelessHART management algorithm and the whole stack were not implemented either. Shah *et al.* [14] implemented WirelessHART based on their previous work on TrueTime [13], and they abstract away from the physical layer of the communication and move toward the application levels and control loops. They did not, however, cover multi-hop and multi-channel communication. The authors in [15] propose the use of a co-simulation framework based on the interaction of TrueTime, together with a cross-layer wireless network simulator based on OMNET++ for improving overall coexistence management.

## 3. WirelessHART Architecture

**Figure 4.**
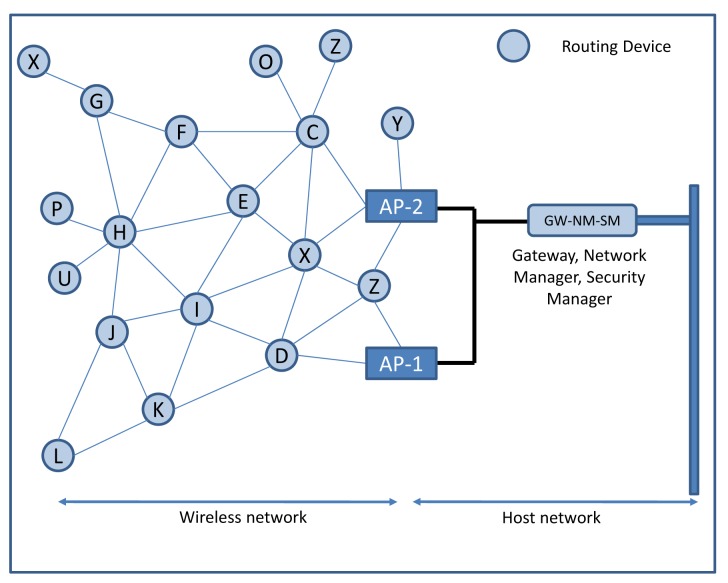
A sample WirelessHART (HART (Highway Addressable Remote Transducer)) network topology. AP : access point.

The WirelessHART protocol has been designed in order to implement a sensor and actuator mesh communication system. A typical topology of a WirelessHART network showing its architecture is depicted in Figure 4. The following types of devices (logical and or physical) operate in the network:

- Security manager (SM), whose task is to handle security issues, e.g., the distribution of encryption keys to the network manager in each network.
- Network manager (NM) per network, which forms the network, handles node affiliation, schedules resources (e.g., defining superframes), configures routing paths, monitors and reports the network health, *etc.* Redundancy can be ensured by using multiple (passive) NMs.
- Gateway (GW), whose task is to interconnect field devices with the plant automation system by exploiting one or more access points.
- Access points are attached to the gateway and provide redundant paths between the wireless network and the gateway.
- Routers are deployed in the network to improve network coverage and connectivity. In WirelessHART, the routing role is usually executed by field devices. However, additional routers can be added to allow for path diversity, depending on plant obstacles. A router is a special type of device that does not possess a process sensor or control element and, as such, is not connected to the process itself.
- Several field devices, *i.e.*, sensors and actuators, that are connected to the process. All these devices are able to participate in routing tasks.

In addition, there are also other devices with wireless communication interfaces, but those are not connected to the process and are installed in the plant field. Examples include handheld terminals used for commissioning and maintenance purposes and so-called “adapters” that connect legacy hardware with the wireless network.

Commercially available devices often embed the GW-NM-SM (Gateway-Network Manager-Security Manager) roles into a single physical device, as shown in Figure 4. Such a centralized approach allows all the computational burdens to be confined to a single device, thereby reducing the costs of field devices. All communication occurs by moving data to/from the gateway through the intermediate routing devices, thereby following the preassigned routing path. This architecture, despite its simplicity, ensures efficiency in a plant network in which nodes are rarely reconfigured or added during the network's lifetime and where network requirements are rather static.

Furthermore, a centralized architecture facilitates the implementation of a wide variety of network topologies, e.g., according to peculiar application requirements. In a high-performance scenario, it is probably better to adopt a star topology (*i.e.*, all devices are one hop away from the gateway). In contrast, a multi-hop mesh topology is useful for a less demanding scenario (from the timing point of view), like monitoring. Any type of intermediate topology, e.g., cluster-tree networks, can also be realized.

## 4. WirelessHART Implementation

As existing implementations of the WirelessHART are rather incomplete, we decided to implement a complete implementation, including the WirelessHART stack, as well as the GW-NM-SM functionalities. The WirelessHART standard does not specify the specific optimization algorithms that can be used by the network manager to allocate resources and to construct the routes. In [16–22], the authors have proposed the centralized scheduling algorithm in WirelessHART for convergecast by considering linear, tree and mesh networks models. The management algorithm described in [16] was selected for the NM. It is one of the few network management algorithms that addresses both routing and communication scheduling.

### 4.1. WirelessHART Protocol Stack

**Figure 5.**
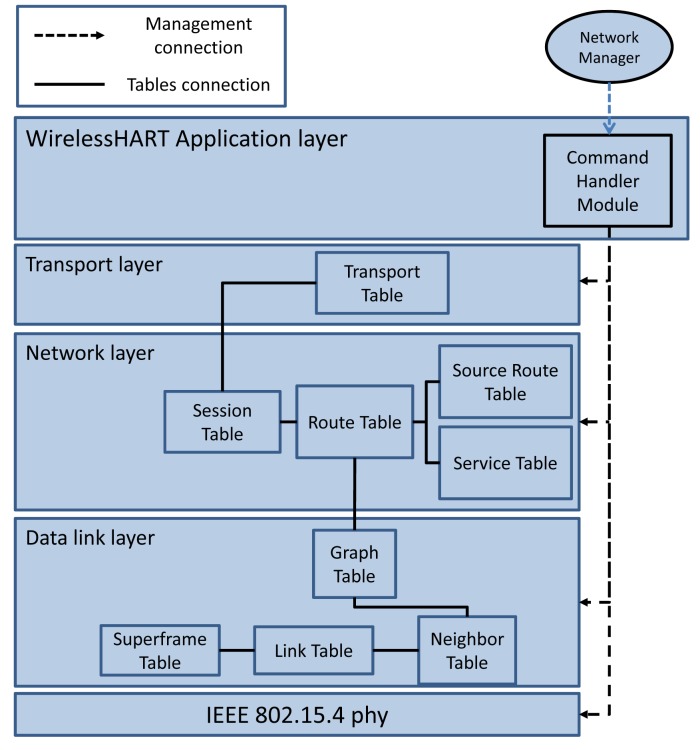
WirelessHART protocol stack.

The WirelessHART protocol stack is shown in Figure 5. All field devices and access points in the network should support this stack.

#### 4.1.1. Physical Layer

The physical layer of WirelessHART is the IEEE 802.15.4 standard's physical layer, which already exists in the WPAN (Wireless Personal Area Network) module of NS-2. We used this layer without modification in our implementation.

#### 4.1.2. Data Link Layer

We modified the MAC layer of the IEEE 802.15.4 standard (2003 version) module available in NS-2 to support network-wide time synchronization, channel hopping, dedicated slotted unicast communication bandwidth, link layer ACKs (Acknowledgements) and concurrent link activation. Several new MAC layer management entity (MLME) primitives, based on the IEEE 802.15.4e (TSCH mode) standard, were also added. The added MLME includes: *mlme_set_slotframe, mlme_set_link, mlme_set_graph, mlme_tsch_mode, mlme_listen, mlme_advertise, mlme_keep_alive, mlme_join, mlme_activate* and *mlme_disconnect* [10].

The communication tables shown in the data link layer of Figure 5 are also implemented. They are manipulated by the NM through the MLME primitives. The tables include:

- Superframe table: This table contains a collection of superframes. Based on the required communication schedule, multiple superframes of different lengths can be configured for each device by filling in this table. The practical superframe length is defined as 2*^n^* s (−2 ≤ *n* ≤ 9) from 250 ms (2^−2^ s) to 8 min and 32 s (2^9^ s) [16].
- Link table: This table contains a collection of links. This table, together with the superframe table, identifies the communication schedule. Based on the traffic rates, multiple links are scheduled for each device in different periods (by specifying the superframe ID to which the link belongs). Each link is specified by the node address, timeslot, channel offset, link type (normal, join, discovery or broadcast) and link option (Tx-link, Rx-link, or shared Tx-link).
- Graph table: In a graph table, each graph lists the potential next-hop neighbors to which the data can be forwarded. This table, in collaboration with the route table located in the upper layer, provides sufficient information for routing the packets.
- Neighbor table: Unlike the other communication tables, this table is not filled by the NM. The neighbor table contains the list of neighbors the device can communicate with.

#### 4.1.3. Network Layer

The network layer provides routing and secure end-to-end communication for network devices in WirelessHART To provide secure communication, a security sublayer is implemented in the network layer itself. As there is no session layer defined in the WirelessHART stack, a session is defined in the network layer. To support graph routing and source routing, the Route Table and Source Route Table shown in Figure 5 are implemented. These tables are manipulated by the NM and are used to deliver a packet to the destination.

##### Sessions

Sessions ensure secure (end-to-end encrypted) communication between two devices in the network (e.g., between the NM and an Input / Output (I/O) device or between the Gateway and an I/O device). Four sessions are generally defined in WirelessHART, and all the devices (including the gateway and NM) support them [1]. These sessions are the following:

- A unicast session between the NM and the device. This session is used to manage and configure the network by the NM.
- A broadcasting session between the NM and all the devices. This session is used to broadcast similar management data to all the devices.
- A unicast session between the gateway and the device. This session is used to publish (or subscribe to) the sensor data between the devices and the gateway.
- A broadcasting session between the gateway and all the devices.

##### Services

In WirelessHART, services are used to allocate bandwidth for a specific type of data. The list of services allocated to a field device is stored in the Service Table shown in Figure 5. In general, four service types are supported by WirelessHART:

- Maintenance and configuration (default): This service is used to give the wireless network a minimum overhead bandwidth for basic network control communications [23].
- Publish: This service is enabled when the device needs to periodically send data or needs to do so on an exception basis. Reporting a sensor reading on a fixed interval constitutes an example of periodic communication [23].
- Block Transfer: This service is used to send large consecutive blocks of data, such as data log files [23].
- Event: This service is used to send data packets during unexpected events, such as warnings. These events normally occur infrequently. However, when they do occur, the delivery of the data packet is usually urgent. The bandwidth services must therefore be established ahead of time [23].

#### 4.1.4. Security Sublayer

WirelessHART provides secure communication between end devices. This is achieved by using cryptographic services in different layers, such as the data link layer and the network layer. We use the Crypto++ library [24], which supports various algorithms. The CCM (counter with cipher block chaining message authentication code (CBC-MAC)) algorithm with the AES (Advanced Encryption Standard)-128 mode of operation is used in our implementation [24].

WirelessHART adopts the CCM* algorithm, which is an IEEE extension to the CCM algorithm. In the IEEE 802.15.4-2006 standard, it is noted that for the CCM algorithm, the sum *L* + *n* = 15 holds, in which *L* is the placeholder for the size of the message to be enciphered and *n* is the size of the nonce. As *n* is 13 in the WirelessHART standard, we get *L* = 2. Since the length of the message integrity code (MIC) is fixed and not equal to zero, there are no constraints for the nonce. Hence, the standard is actually using just CCM instead of the CCM* mode.

**Figure 6.**
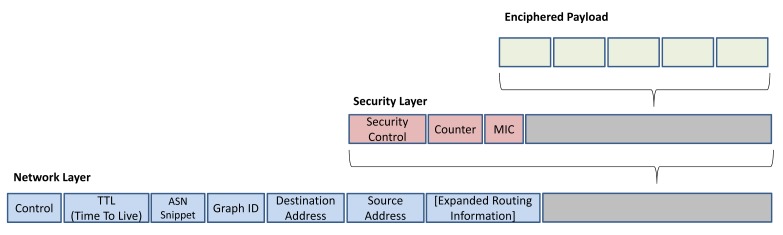
The WirelessHART network layer data unit structure. MIC: message integrity code; ASN: Abstract Syntax Notation.

At the data link layer, the integrity of the messages is checked by calculating the MIC in the data link layer, to see if the packets are received from the valid sender. In addition, at the network layer, the security sublayer checks the integrity of the messages that travel several hops to the destination by calculating the MIC. The network layer protocol data unit (NPDU) is shown in Figure 6. The security control byte indicates the type of security, which can be session keyed, joined keyed or handheld keyed for each packet [1].

In order to let the intermediate routers forward the packet to its final destination, the NPDU header is not enciphered. Therefore, only the NPDU payload is enciphered to ensure reliable communication. To authenticate the NPDU and to decipher the NPDU payload, a keyed MIC is added to the security sublayer. The MIC ensures secure communication by checking whether the NPDU received from the correspondent node is forged or not. The CCM* mode is used to generate the MIC, in conjunction with the AES-128 block cipher. At the final destination, the AES-128 engine authenticates the received packet and deciphers the payload.

#### 4.1.5. Transport Layer

The transport layer ensures that packets are delivered successfully across multiple hops to their final destination. This layer supports either acknowledged or unacknowledged transactions. Unacknowledged service is used for delivering packets that require no end-to-end acknowledgment, e.g., sensor data publishing. On the other hand, the acknowledged service is used to deliver packets that require confirmation of their delivery. The field devices act as slaves during unicast and broadcast communications from the NM or gateway; but, they act as masters (publisher) when sending event notifications to the NM or gateway, as well as during service request procedures.

For each acknowledged transaction, a new entry is created in the Transport Table shown in Figure 5. A transport pipe that connects two devices is constructed across the network. Each WirelessHART device might track multiple transport pipes. The gateway and the NM often track many transport sessions with each field device. For example, when it uses the acknowledged broadcast initiated by the gateway or NM, the transport layer tracks the reception of acknowledgment from all the affected devices. The transport layer also supports the aggregation of multiple HART commands in a single transaction. This method is especially useful when sending (or reading) several configuration commands to (or from) a network device.

#### 4.1.6. Application Layer

The application layer of WirelessHART is a command-based layer. Commands, the basis of HART communications, are sent from the gateway or field devices. Each command can be identified by a command number, which determines the content of the message. The WirelessHART commands are a collection of commands in the range 768–1023, which can be used to support network management and gateway functions [1]. The commands implemented can be classified into the following categories: managing superframes and link commands, managing graph and source route commands, bandwidth management commands, network health reporting and status commands.

### 4.2. WirelessHART Network Management Algorithm

The WirelessHART NM uses centralized network management techniques for communication scheduling and managing routes. However, it does not define any specific algorithm for the NM. The management algorithm introduced in [16] is one of the few network management algorithms that address both routing and communication scheduling. We choose this algorithm for our implementation. According to [16], each time a new node joins the network, the algorithm is executed, and it tries to find new uplink, broadcast and downlink graphs and defines communication schedules for the new device. This process is done incrementally, until all the nodes join the network.

This section considers the implementation of the network management algorithms, by discussing their four most important parts: the joining procedure, graph and route definition, communication scheduling and, finally, the service request procedure.

#### 4.2.1. Joining Procedure

**Figure 7.**
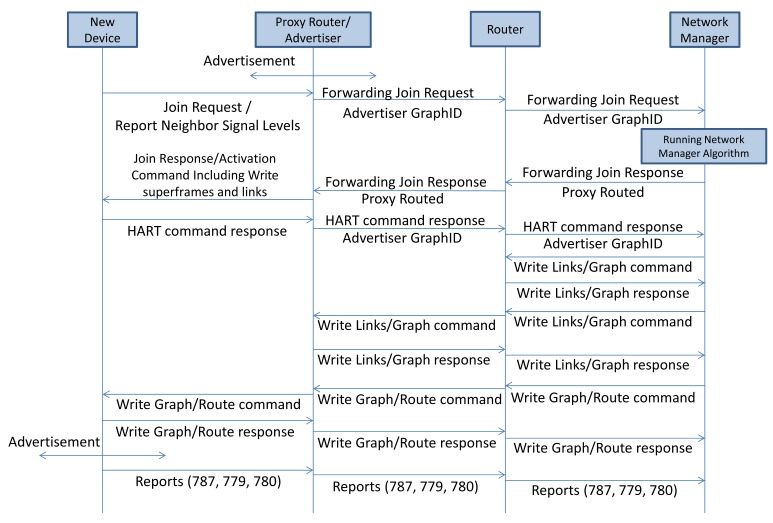
Joining process.

The joining sequence of a new device is shown in Figure 7. Nodes that have already joined the network periodically send advertisements used for synchronization purposes and to inform nodes that want to start the binding process about the superframes' structure. Nodes that want to participate in the network must know the (time) position of the join time slots in the superframe; in these join time slots, nodes are allowed to send join requests. The new device that intends to join the network listens consecutively on all physical channels for a while. It selects the best advertiser/candidate based on certain predefined criteria and sends the join request to the selected advertiser. The join request contains report neighbor signal levels (Command 787), as well as other information. The new device includes the advertiser graph ID in the network header. The join request is forwarded toward the gateway/NM. The NM allocates network resources (such as graphs and links) based on the management algorithm upon receiving the join request. The NM sends a join response/activation command to the new device, after all necessary network resources are configured and reserved along the path. The NM then sends the join response, including three commands, write network key (Command 961), write device nickname address (Command 962), and write session (Command 963). Finally, the NM sends the commands to write the superframe and links in the communication table of the new device. These are the only commands, besides the join response, that can be proxy routed.

#### 4.2.2. Graph and Route Definition in the Network

To address different communication requirements, three types of routing graphs are defined in any WirelessHART network.

- The uplink graph is a graph connecting all devices to the gateway. It is used to forward both the devices' management data and process data to the gateway.
- The broadcast graph connects the gateway to all devices. It can be used to broadcast either common data or control data to the entire network.
- The downlink graph is defined per device. It is used to forward unicast messages from the gateway to each individual device.

To construct these graphs in a reliable manner, the algorithms “Constructing Reliable Broadcast Graph”, “Constructing Reliable Uplink Graph” and “Constructing Reliable Downlink Graphs” in [16] are implemented in the NM. These algorithms are designed to maintain the maximum number of reliable nodes in the graphs while achieving good network latency.

#### 4.2.3. Communication Scheduling and Channel Management

**Figure 8.**
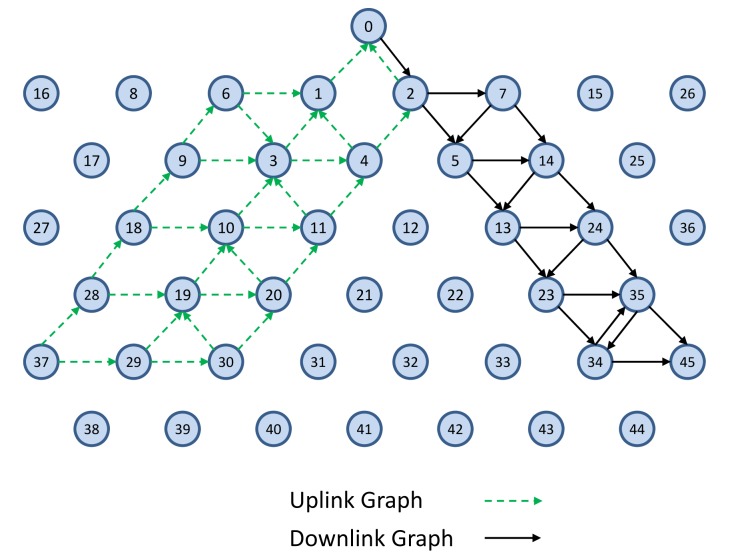
A sample connection establishment between Nodes 37 and 45.

After constructing the uplink, broadcast and downlink graphs, the algorithms “Constructing Data Communication Schedule” and “ScheduleLinks” in [16] are used to construct the data communication schedules and to define links and superframes. These algorithms are implemented in the NM. These algorithms use the fastest sample rate first policy (FSRF) to schedule the devices' periodic publishing and control data. The construction is based on reliable graphs. In Figure 8, a sample connection is shown in which the NM has allocated the resources from the sensor node (37) to the actuator node (45). The sensors publish process data using Commands 1, 3, 9, *etc.* Command 79 is used to write data to the actuators [25]. In this work, similar to what is described in [25], we assume that WirelessHART supports control in the host or control in the gateway.

#### 4.2.4. Service Request Procedure

**Figure 9.**
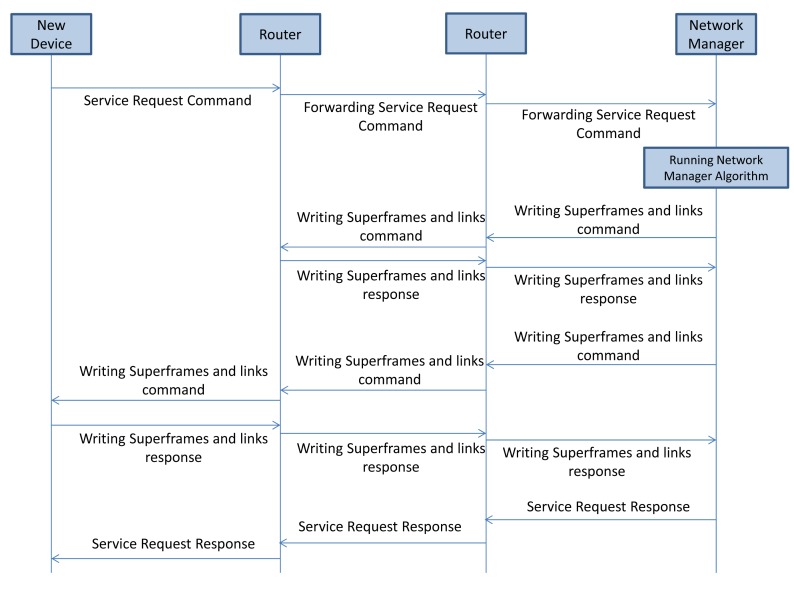
Service request process.

A device that needs to establish a connection with the other devices, e.g., actuators, sends out a service request (Command 799) to the NM asking for additional bandwidth. The service request handling procedure is illustrated in Figure 9. The NM allocates sufficient bandwidth along the uplink graph from the sensor to the gateway and along the downlink graph from the gateway to the actuator, by adding links in a new route or an existing route. This process may take some time. Upon completion, the NM replies to the requesting device.

## 5. WirelessHART Validation

**Figure 10.**
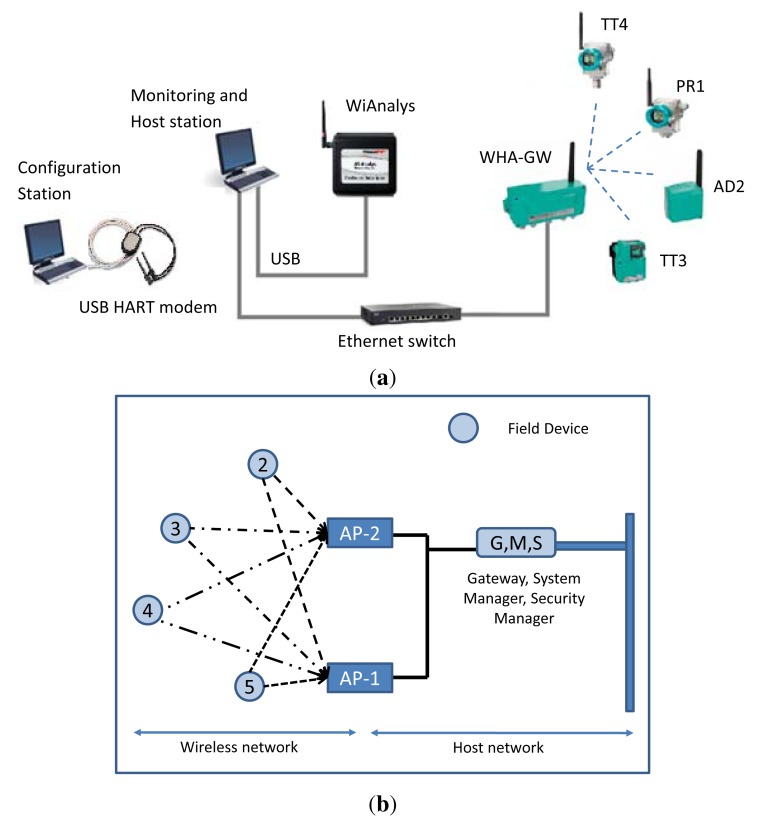
Network topology for (**a**) real and (**b**) simulated setups. GW: gateway.

To validate the WirelessHART simulator implementation, we need a real WirelessHART network to generate the traffic patterns. A testbed has been purposely designed in order to emulate a typical industrial environment, *i.e.*, an instrumented steam generation process at the Idrolab of ENEL in Italy. A similar network has been set up in the NS-2 simulator. The network topologies of real and simulated setups are shown in Figure 10, in (a) and (b), respectively. The collected traffic from the real network is used to (i) validate the correctness of the implementation of the WirelessHART stack and (ii) to confirm that the NM used in the simulator manages the network in a similar fashion as the real NM used in industry.

### 5.1. Real World Experimental Setup

**Figure 11.**
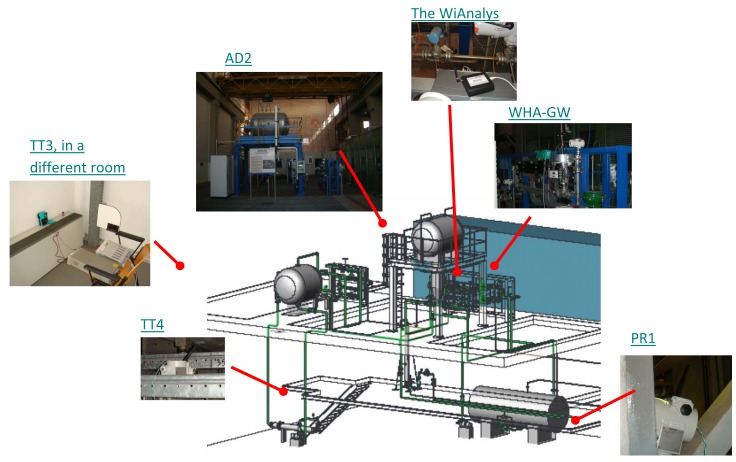
Idrolab test plant.

The Idrolab test plant [26] is depicted in Figure 11. The instruments are not actually attached to the plant, but they flank the legacy of existing wired control systems in order to experience similar harsh environmental conditions. This WirelessHART network comprises:

- A PC-based host station implementing a Mobus/TCP and an OPC (OLE for Process Control) client, both of them purposely implemented in LabVIEW (Laboratory Virtual Instrument Engineering Workbench). The Modbus/TCP server is embedded in the WirelessHART gateway, while the OPC server is implemented by means of the HART server, which translates OPC messages into HART/IP requests and responses. In addition, the PC can directly inject HART/IP traffic into the network.
- A WirelessHART gateway with GW-NM-SM functionalities from Pepperl+Fuchs (WHA-GW) based on the Dust Networks' SmartMesh IA-510 device. It provides an Ethernet connection towards the host application, supports HART/IP and Modbus/TCP protocols and handles Modbus RTU (Remote Terminal Unit) (not used in this work).
- A pressure transmitter from Siemens (Sitrans P280, PR1, Bavaria, Germany).
- A WirelessHART adapter from Pepperl+Fuchs (WHA-ADP, AS2), which can acquire the signal coming from a legacy 4–20 mA transmitter.
- A temperature transmitter from P+F (WHA-UT, TT3); the actual sensing element is an external PT100.
- A temperature transmitter from Siemens (Sitrans TF280, TT4); the actual sensing element is an external PT100.
- A PC-based monitoring station tool to collect data exchanges over the Ethernet link and over the air, implemented by the host station PC.
- A PC-based configuration station used to commission the network leveraging on a USB HART modem from Microflx for field devices' network ID, join key and operating parameters.

We need to collect the traffic patterns from the real network to be able to validate the simulator. We use the open source tool, Wireshark, to analyze the Ethernet traffic. Regarding the over-the-air traffic, two possible approaches can be practically adopted based on the channel hopping mechanism. In the first one, the blacklisting feature offered by the WirelessHART protocol can be exploited to limit the number of radio frequency channels used for hopping without losing generality. For instance, one can use this feature to limit the radio channels to a subset and use a reduced number of low-cost IEEE 802.15.4-compliant protocol analyzers. For example, we exploited three low-cost USB connected radio probes UZBee devices from Flexipanel [27]) to collect traffic logs from three active channels (formally, 22, 23 and 24). These three traffic logs were then merged, based on the collected messages' timestamps.

The second approach exploits fifteen transceivers (WirelessHART only supports fifteen channels) to simultaneously scan all the ISMband at 2.4 GHz. For instance, we also use the the WiAnalys tool, developed by the HART Communication Foundation (HCF) consortium [28], that hosts an FPGA (field-programmable gate array) for managing the IEEE 802.15.4 transceivers. In both cases, post-process software running on the monitoring station decodes raw message logs and recognizes different stack levels. The results presented in this paper refer in particular to the WiAnalys tool.

#### 5.1.1. Addressing Security Aspects

During the data collection from the real network, there is a need to address the security authentication in the data link layer and to decrypt the NPDU. We manage to authenticate the messages with the MIC. To calculate the MIC of the DLPDU (Data-Link Protocol Data Unit (*i.e.*, a Data-Link Layer packet)) during the joining process, we use the well-known public key, 7777 772E 6861 7274 636F 6D6D 2E6F 7267 hexadecimal, which is the ASCII value sequence of the 16 character string of the HART Foundation's web address: www.hartcomm.org. We decrypt the NPDU from the join request message using the join key of each device, which is known in advance. After the successful decryption of the join requests, we follow the initialization command, which contains the new session keys that will replace the join key. We also follow the new network key, which will replace a well-known key for calculating the MIC at the data link layer for each I/O device. Each following message is then first decrypted and checked if it contains the command for changing the session keys or the network key, in which case, we save the new keys for that particular node and start using them with the next message. The authors developed the procedure of extracting the session key in the simulator.

### 5.2. Simulation Model and Parameters

**Figure 12.**
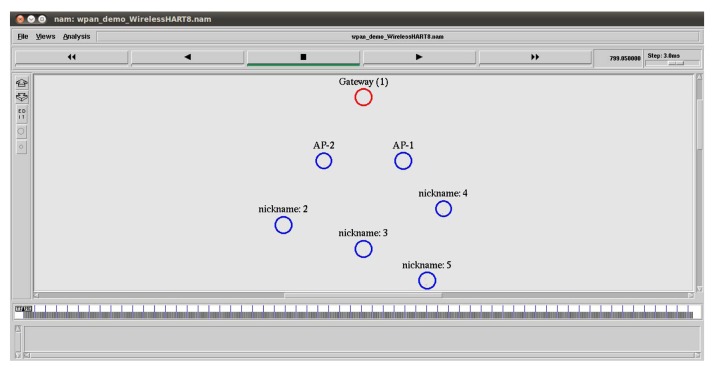
The network topology from the animation tool of the NS-2 simulator (NAM).

In the NS-2 simulator, we set up a similar network with four field devices, which are connected to the gateway through two access points (APs), as shown in Figure 12. It is a snapshot taken from NAM (Network Animator), the Tcl/Tk-based animation tool for viewing network simulation traces in NS-2. We assume that the connection between APs and the gateway is wireless. The details of the simulation parameters are presented in Tables 2 and 3. We choose the shadowing radio propagation model, as it is a more general model allowing for more realistic predictions with multi-path and fading effects [29]. The shadowing model consists of two parts, as shown in Equation (2). The first part is the path loss model that predicts the mean received power at distance *d* and *d*_0_ as a reference-distance, while the second part reflects the variation of the received power, which is a Gaussian random variable with zero mean and standard deviation *σ_dB_*. *σ_dB_* is referred to as a shadowing deviation, and its value for two different environment are provided in Table 2.

(2) $${\left[\frac{P_{r}(d)}{P_{r}(d_{0})}\right]}_{dB}=-10\beta \log\left(\frac{d}{d_{0}}\right)+X_{dB}$$

The simulation scenarios are implemented in NS-2, by using Tcl scripts. The scripts comprise commands and parameters for simulator initialization, node creation and configuration, such as *startWHGateway, startWHAccessPoint, startWHDevice* or *requestService* commands. The commands can be used respectively to start a gateway/NM, access points, field devices and to request more bandwidth to communicate with the other devices.

### 5.3. Validating the WirelessHART Stack

To validate the WirelessHART stack implemented in the simulator, the simulated NM is replaced by the real NM. The radio frequency and the time stamp at which the sniffer receives the packet identify each packet in the traffic log file. All traffic generated by the real NM is filtered from the traffic log file. This traffic includes the joining response, activation commands and all management commands that manipulate the communication table, as well as different tables in the field devices. For this traffic, a virtual NM generates corresponding events in the simulator at the same frequency and time. Thus, the times of the simulator and the real network get aligned. The neighboring field devices of the virtual NM receive the packets and forward them to the destination node. At the destination node, the packet traverses through each layer of the simulated stack and reaches the command handler in the application layer. By checking the validity of the commands received, it is possible to verify the implemented WirelessHART stack.

### 5.4. Validating the WirelessHART Network Manager

In order to validate the simulated WirelessHART NM, we need to show that the implemented NM manages the network similar to the real NM. To this end, we create a network with the same number of field devices in the simulator as there are in the real test-bed scenario. By measuring the management overheads, reliability, end-to-end delay and communication scheduling of both the simulated network and the real network and by comparing the collected statistics, we show that the two NMs function almost in a similar manner, and thereby, we can validate the simulated WirelessHART NM. The details are described in Section 6.

## 6. Experimental Analysis of Real and Simulated Networks

We collect traffic patterns from the real network installed at the testbed in the Idrolab for about 24,000 s. Initially, all field devices are placed within 40 m from the gateway, and they form a star network with the gateway. After some time, Node 5 is moved away from the gateway, so that a two hop network is formed. In the simulator, we consider a similar placement, but with a fixed position for Node 5, after which a star network is formed. Since Node 5 is located far away from the access points, as shown in Figure 12, Node 5 sometimes uses Node 4 as an intermediate node, and a two hop network is also formed.

### 6.1. Reliability in the Network

**Figure 13.**
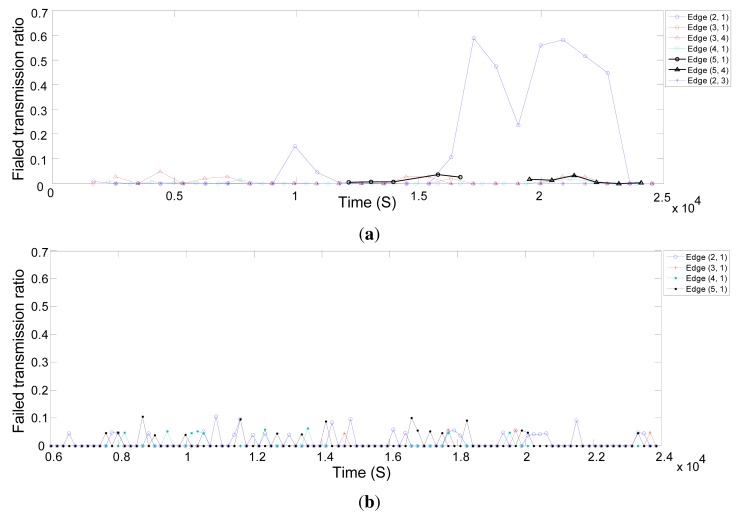

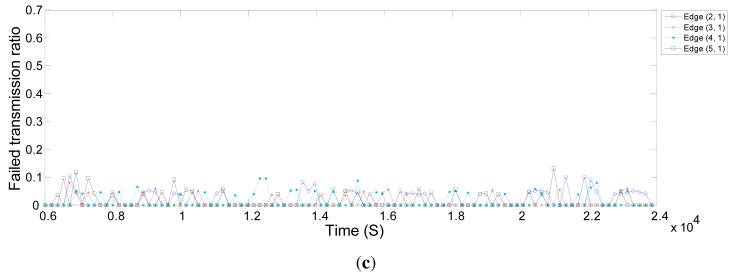
The failed transmission ratio on different edges over time. (**a**) Real network; (**b**) Simulated network with a shadowing deviation of 5.7 dB; and (**c**) Simulated network with a shadowing deviation of 8 dB.

In this section, we evaluate the behavior of the real network and the simulated network in terms of reliability. We use the neighbor health list report to evaluate the quality of connections between the network field devices. These reports provide the statistics for linked neighbors. Figure 13a shows the percentage of failed transmissions on different edges in the real network over time. A very small percentage of transmissions fails, except for the edge (2,1) between Node 2 and the gateway. When the connection quality drops between Node 2 and the gateway, the NM defines more links between Node 2 and Node 3. Some of the traffic of Node 2 to the gateway is forwarded through Node 3. As a result, the problem is fixed. Figure 13b,c shows the percentage of failed transmissions on different edges in the simulated network over time, with shadowing deviations of 8 dB and 5.7 dB that correspond to the corridor and engineering building [30] environments, respectively. As the deviation increases in the shadowing model, the packet drop increases likewise.

**Figure 14.**
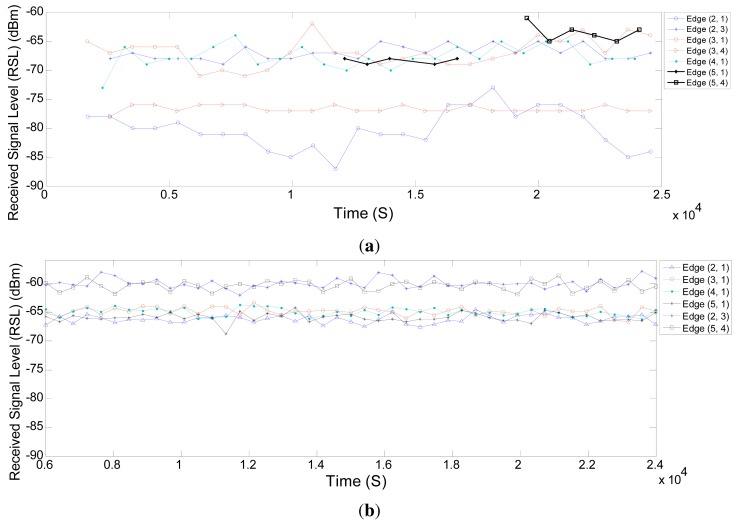
The average of receive signal level (RSL) on different edges in the network over time (**a**) for a real network and (**b**) for a simulated network.

Figure 14a,b displays the average of receive signal levels (RSL) on different edges in the real and simulated network over time. RSLs considerably differ from one another in real networks, whereas in simulations, they are quite close. We also see in Figure 14a that the RSL between Node 2 and the gateway varies a lot over time. This variation also justifies the earlier mentioned statement that the NM defines more links between Node 2 and Node 3 to overcome the problem in the connection between Node 2 and the gateway. For the connections between other nodes and the gateway, the RSL values in the simulation are very close to the real values. They deviate between −65 dBm and −70 dBm.

### 6.2. Communication Schedules and Network Throughput

**Figure 15.**
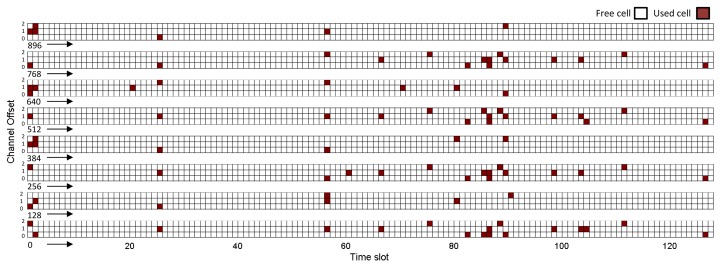
The global matrix of the current slot/channel usage for the real WirelessHART network (the combination of superframes with sizes of 128, 256 and 1,024 time slots).

**Figure 16.**
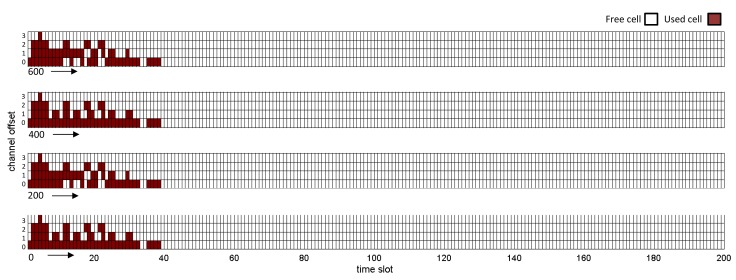
The global matrix of the current slot/channel usage for the simulated WirelessHART network.

Figure 15 and 16 show the global matrix of the reserved communications by the NM in the real and simulated network scenarios. The real network has a combination of superframes with sizes of 128, 256 and 1,024 time slots, whereas the simulated network has a superframe length of 200 time slots. The NM schedules interference-free cells to transmit management traffic or sensor data. We can see that the allocation patterns are quite different. This is because the simulated NM constructs the communication schedules based on the proposed algorithm in [16], where it allocates from the source to the destination each link on the paths in a depth-first manner. Hence, it allocates the earliest available timeslot to each link and updates the schedule matrix, as well as each effected node's schedule accordingly. In the real NM, the undisclosed algorithm seems to allocate cells randomly. Since the simulated NM allocates more links between devices, the communication schedule in Figure 16 is denser than the communication schedule in Figure 15. Allocation of more cells might affect management efficiency due to: (i) the joining process delay and overhead; (ii) the bandwidth allocation based on service requests; and (iii) coping with node/edge failure in the network. It might also affect power consumption and end-to-end latency. In such a case, allocating more cells (over-provisioning) will increase the energy consumption of the nodes. On the other hand, it will improve end-to-end latency.

### 6.3. Real-Time Guarantee

**Figure 17.**
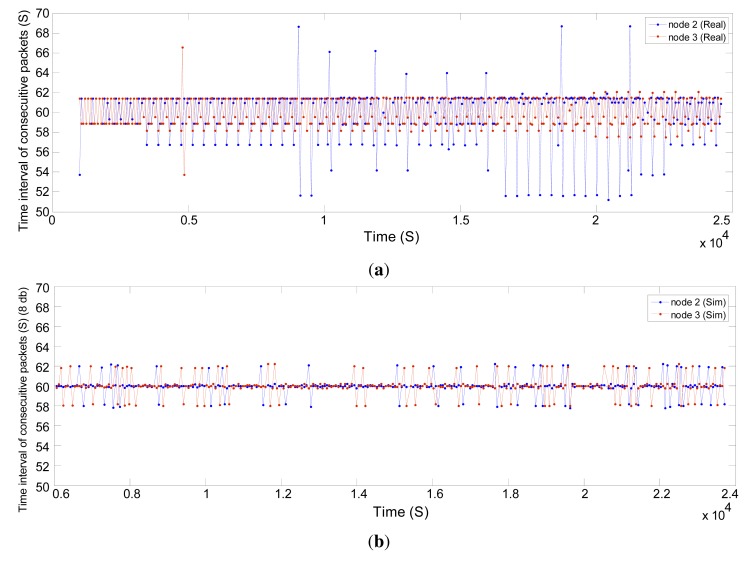
Time interval of the consecutive received packets for Nodes 2 and 3. (**a**) Real network; and (**b**) simulated network with a shadowing deviation of 8 dB.

**Figure 18.**
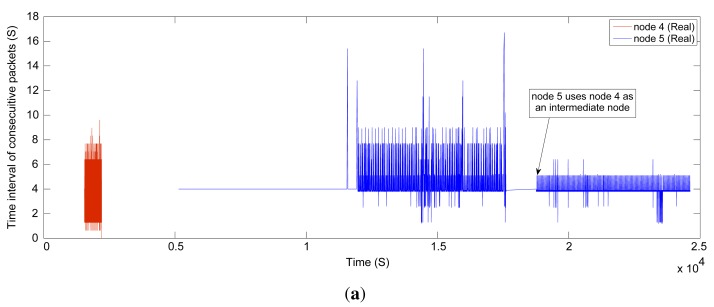

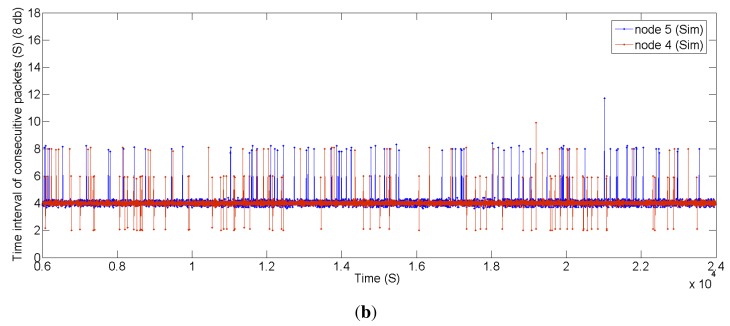
The time interval of the consecutive received packets for Nodes 4 and 5. (**a**) Real network; and (**b**) simulated network with a shadowing deviation of 8 dB.

To evaluate the end-to-end data delivery delay, we measure at the gateway the time interval between the consecutive received packets, which are sent by the field devices during the network operating time. Figure 17a,b displays the results for Nodes 2 and 3 with a constant publishing period of 60 s in the real and simulated network, respectively, while Figure 18a,b shows the results for Nodes 4 and 5 with a period of four seconds. The simulated sensor nodes publish the data at the specified rates following the constant bit rate (CBR) traffic model employed in NS-2. The required resources to support these traffic characteristics are reserved beforehand, along the path between the sensors and the gateway, for both real and simulated networks.

The results show that in the simulator, the real-time communication requirements are addressed much better than in the real network. The presence of external interference in harsh industrial environments could explain this difference. This causes more packet drops and possibly more retries at the MAC layer in the real network.

In Figure 17a, we see that the connection quality (timeliness) between Node 2 and the gateway drops after some time, while the end-to-end delay increases. Then, the NM, at around 2.1 × 10^4^ s, defines more transmission links between Node 2 and Node 3, and some of the traffic of Node 2 toward the gateway is forwarded through Node 3, bringing down the end-to-end delay. In addition, we see in Figure 18a that the connection quality between Node 5 and the gateway drops after a certain time. This is caused by an intentional increase of the distance between these nodes in the real network. At around 1.8 × 10^4^ s, the NM considers Node 4 as an intermediate node between Node 5 and the gateway and writes several links between Node 4 and Node 5. Afterwards, the end-to-end delay is reduced significantly. In the simulation, we did not move Node 5, and so, no such variations are seen.

In an industrial environment, we expect large shadowing, due to the presence of heavy machinery, which typically causes a positive biased shadowing effect. The shadowing effect can vary according to different industrial setups. In the simulator, we choose the shadowing model. In order to simulate a harsh industrial environment, we need to propose a channel model that represents that environment more accurately.

### 6.4. Energy Consumption in the Network

In this section, we evaluate the energy consumption of network nodes in real and simulated scenarios. We measure the total consumed energy at every node during the 24,000-s time period of the network operation. The periodic management messages generated by each device in the WirelessHART network consist of network health reporting and status commands (*i.e.*, WirelessHART Commands 779, 780 and 787) and advertisements. Management and application data messages for WirelessHART are listed in Table 3.

The specific values of the parameters used in the calculations are listed in Table 4. The timing parameters are illustrated in Figure 2. Table 5 shows the energy consumption required for each type of transaction (in this calculation, we assumed the energy consumption in Tx/Rx turnaround, and the processing energy can be neglected). In addition, the idle listening energy at an unused scheduled link is calculated: the energy consumed by the receiver while waiting for a message.

Table 6 also lists the energy consumed by each node, as well as by the gateway in both the real and the simulated network. In the simulated network, the energy consumed by the nodes is more than in the real network. Part of this difference can be explained by the fact that in the simulator, we defined more links between the nodes. Furthermore, the considered management message rate is different in the simulator. We also see that the energy consumed by Node 4 is higher than the energy consumed by the other nodes in the simulator. This is because Node 4 is considered an intermediate node in the uplink and downlink graph for Node 5, as it is located far away from the access points.

### 6.5. Evaluating Management Efficiency

In this section, we evaluate the I/O device joining procedure, as well as the service request procedure by measuring the delay and communication overhead in both the real and simulated WirelessHART networks. The network management algorithm greatly affects the performance of the WirelessHART network. Consequently, depending on the network management algorithm that is used, the results discussed in the section may differ.

#### 6.5.1. Performance During Node Joining

In WirelessHART, as discussed in Section 4.2.1., the joining process includes scanning the channels for a while for router discovery, sending the join request to the routers and receiving the management communication resources and related graphs/route information. As shown in Figure 7, the joining process is considered to start from the moment that the node sends the join request till the moment that it begins to broadcast the advertisements and send the periodic reports toward the NM. However, in our comparison of the joining process in simulated and real networks, we consider the total delay and overhead of the management resources reservation without accounting for scanning delay.

**Figure 19.**
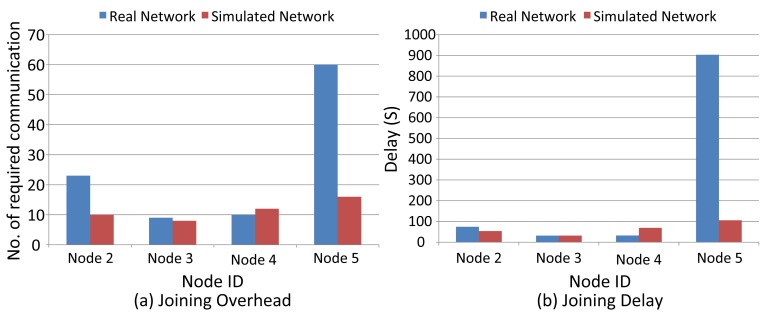
Field device joining overhead (**a**) and delay (**b**) (real *vs.* simulated WirelessHART network).

Figure 19a,b displays the delay in and the number of communications required (number of messages sent) for I/O device joining. There is no considerable deviation in delay and overhead in both scenarios except for Node 5, whose position has been changed in the real experiments.

#### 6.5.2. Service Request Procedure between I/O Devices and Gateway

**Figure 20.**
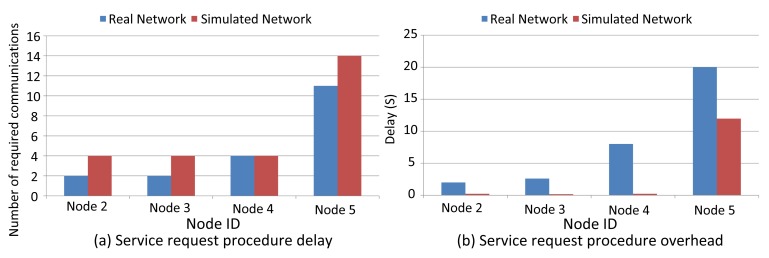
Service request procedure (**a**) overhead and (**b**) delay (real *vs.* simulated WirelessHART network).

In this evaluation, we compare the management efficiency of service request procedures by measuring the delay and the number of communications required for reserving communication resources between field devices and the gateway. Figure 20 shows that for Nodes 2 and 3, the NM does not allocate any communication resources in the real scenario, as it defines sufficient resources during the network setup. The overhead of all nodes in the simulator exceeds the one in the real scenario. This is because the NM assigns more links in the simulator (Section 4.2). Hence, more messages are sent in the simulator than in the real network. This also makes the delay in simulations much lower than in the real scenario, except in the case of Node 5, as links are already assigned. Node 5 uses Node 4 as the intermediate node in the simulation. In the real scenario, Node 5 initially communicates directly with the gateway, but after it has been moved further away from the gateway, the NM considers Node 4 as an intermediate node for Node 5 and allocates new resources between Nodes 4 and 5. This increases the overall delay.

### 6.6. Summary

The evaluation results from the real network randomly deviate, due to the industrial environment, whereas they are more or less consistent in the simulation. In addition, we found that the network management algorithm greatly affects the performance of the WirelessHART network, namely during node joining, the service request procedure, data delivery latency and when coping with node/link failure. Consequently, when applying other system management algorithms, the results may differ.

## 7. Experimental Analysis of a Multi-Hop Mesh Network in the Simulator

**Figure 21.**
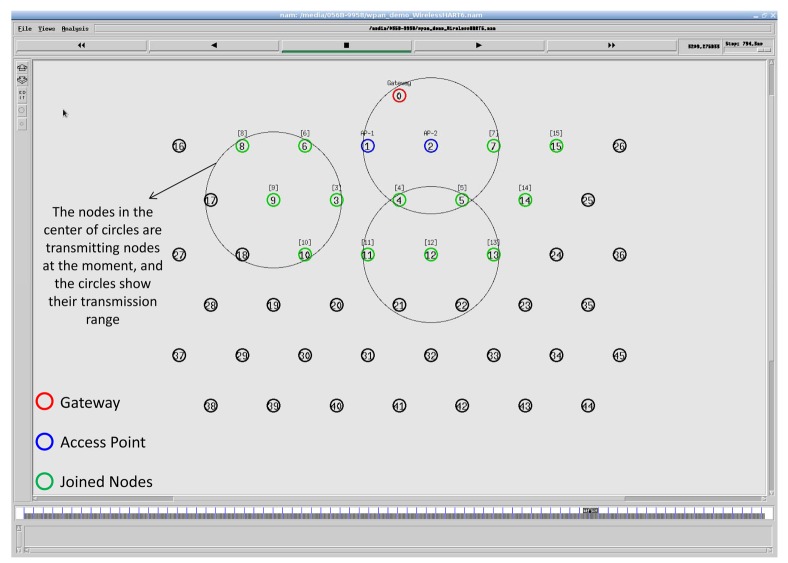
A sample multi-hop mesh network topology in the NS-2 simulator.

In this section, we show some results from a multi-hop mesh network that is used to demonstrate the usability of the simulator. In these experiments, we consider a simulation area of a size of 150 m × 150 m, with field devices placed away from each other at a distance of 10 m, as shown in Figure 21. The transmission range is set to approximately 15 m. We use the two-ray ground model as a radio propagation model [29]. The network consists of one gateway, two access points and 43 field devices. All the results reflect the average values achieved after the experiments were repeated several times.

### 7.1. Node and Link Failure in the Network

**Figure 22.**
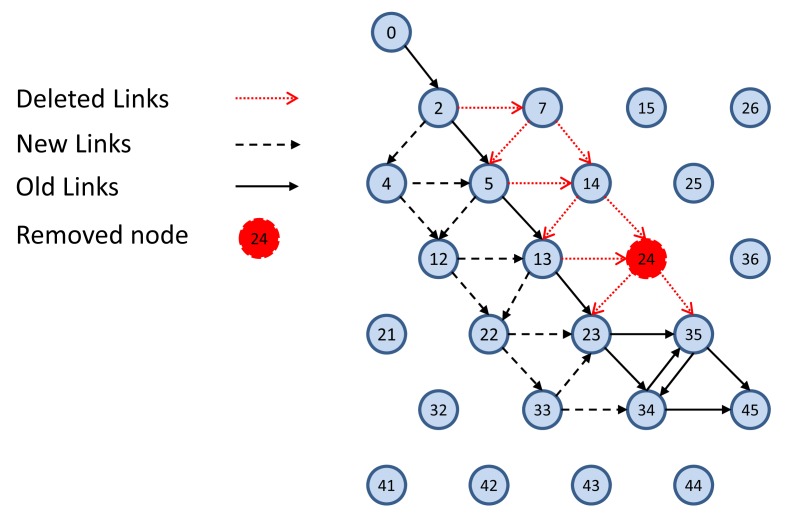
Node failure sample.

In this part, we demonstrate the behavior of the system in case of link and node failure. Figure 6 shows a sample downlink graph toward Node 45. Figure 22 shows the failure of Node 24 in the network, as well as how the system manager copes with this node failure by defining new links and by deleting unnecessary links.

**Figure 23.**
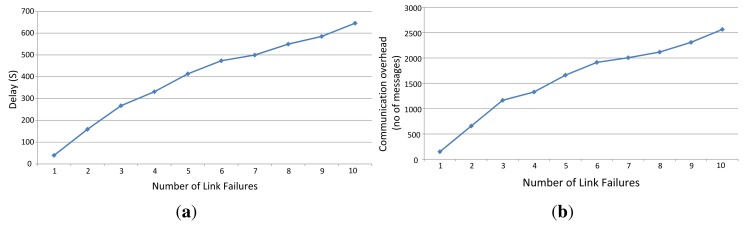
Network maintenance (**a**) delays and (**b**) overhead.

Figure 23a,b shows how the system behaves while link failures are being varied. The link failures are introduced randomly on different hop levels. We increased the number of link failures from one to 10 and measured the delay in and the number of required communications for coping with the link failures. Even though the network may still work when the graphs are unreliable, the implemented management algorithm tries to establish a reliable graph and to construct a new schedule. This causes a relatively high delay and a large number of communications.

### 7.2. Data Delivery Ratio in Case of Lossy Network

**Figure 24.**
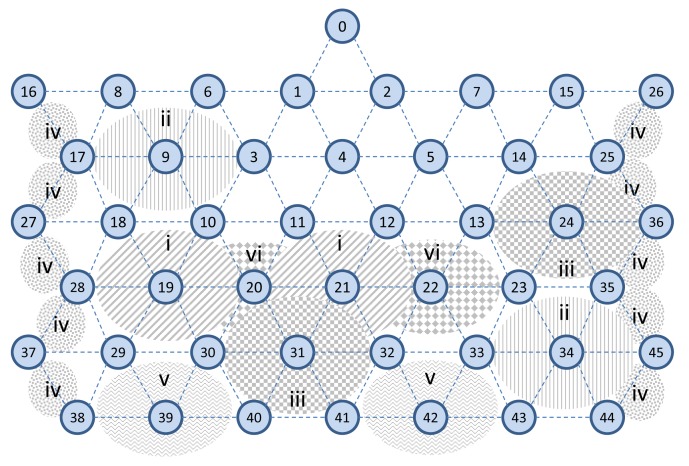
Applying interference incrementally in six steps.

In this experiment, we evaluate the relationship between the packet delivery ratio and increased interference in the network. To do so, we forward the CBR traffic (periodic sensor data) from sensors towards actuators, for 29 end-to-end connections. At the destinations/actuators, we then measure the number of received packets. We assume that the quality of edges may vary from 0% to 100%. We increase the interference regions in the network in six steps (each step takes 2,000 s), as shown in Figure 24. In each region, we randomly apply a different interference value to the edges between the nodes, so that each device may experience a different packet loss ratio, as is the case in real harsh environments.

We evaluate the performance in three scenarios. The first scenario (WH s1: WirelessHART Scenario 1) is the worst-case scenario, in which the WirelessHART NM is configured in such a way that it will not adjust the affected routes, after receiving the path alarms commands and neighbor statistics reports form the network. In this scenario, each node randomly selects its next hop based on the WirelessHART protocol. In the second scenario (WH s2: WirelessHART Scenario 2), we assume that the condition of the poor (interfered) edges will be reported to the NM by neighbor health list reports, conforming to the WirelessHART protocol. The NM re-establishes new graphs through the least affected edges, releases the previously reserved resources on the old path, and then reserves new resources along the new graph/route. These instructions are forwarded towards the network devices. Each node selects the next hop neighbor on the new given graph in a random manner, in line with the WirelessHART protocol. In the third scenario (WH s3: WirelessHART Scenraio 3), similarly to the second scenario, we assume that the NM re-establishes new graphs. However, to pursue better data delivery ratios, each node chooses the best next hop neighbor based on local information (an action that is not listed in the WirelessHART protocol) on the packet loss ratio of each neighbor.

**Figure 25.**
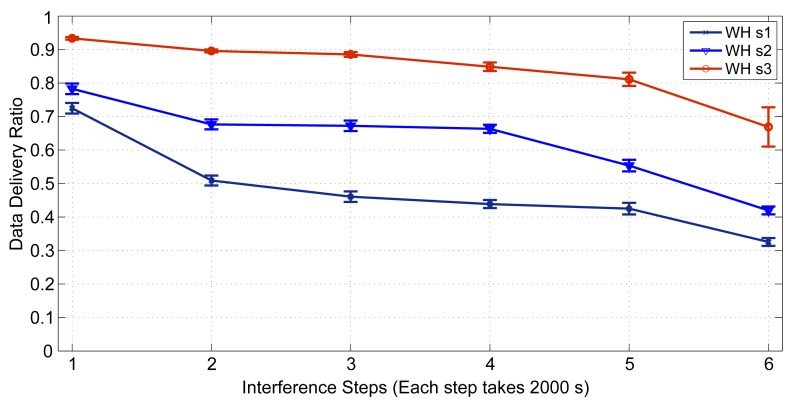
Data delivery ratio differences in three scenarios.

We see in Figure 25 that WH s3 performs much better than WH s2, which, in turn, performs better than WH s1. The poor performance in the WH s1 can be expected, as the NM does not adjust the affected routes. The large differences between WH s3 and WH s2 can be explained by the fact that in WirelessHART, more edges are defined in the uplink and downlink graphs to increase their reliability. If the interference in question is extensive in the network, the repaired graphs still may include some poor edges, as well. Therefore, if the nodes randomly choose the next hop, these poor edges may also get selected, and the network performance decreases.

The results also confirm that it is quite easy to evaluate the impacts of the changes and enhancements that we make, as long as a simulator is readily available.

**Figure 26.**
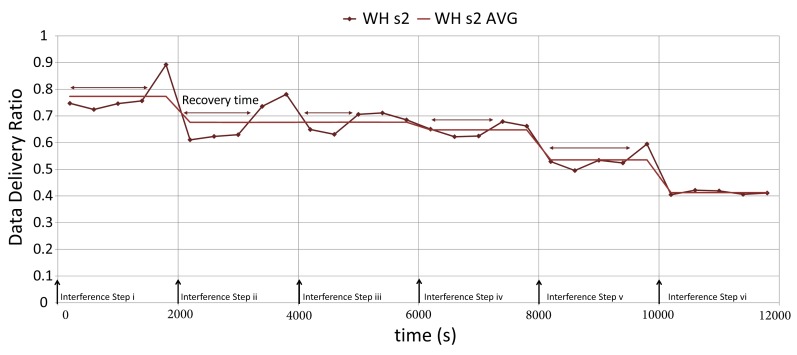
The data delivery ratio difference in two scenarios.

In Figure 26, we consider five measuring points in each step to have a more detailed view of data delivery ratio changes between interference steps. We consider only WH s2, the actual scenario in WirelessHART. The data delivery ratio suddenly drops each time the interference is applied in the network. As expected, WirelessHART requires quite some time to reach a stable data delivery ratio value. In this figure, the WH s2 AVG (Average) represents the average value of the data delivery ratio in each step.

## 8. Usage of WirelessHART Implementation

In this section, we provide some examples of usages of the WirelessHART simulator that can help network/control engineers, as well as protocol designers.

### 8.1. Feasibility Study of WirelessHART in Different Application Scenarios

Network engineers can check the feasibility of using the WirelessHART network in various application scenarios with different requirements very easily with our implementation. By having predefined application scenarios, *i.e.*, the number and position of nodes, as well as the sampling rates of sensor nodes, they can study whether the NM can allocate sufficient resources/bandwidth in those scenarios or not. The network designers can also study the network coverage and connectivity characteristics. For example, by considering the network topology in the NM, additional routers might be deployed in the network, if the sensor and actuators are not covered or if the constructed graphs are unreliable. The control engineers can also study the possible data delivery delay in the control loop.

By using the Tcl scripts, such as *WHnode-down, WHlink-down, WHnode-up* or *WHlink-up*, which the WirelessHART simulator provides, network designers can simulate node/link failure and introduce dynamicity into the network. They can evaluate whether the WirelessHART protocol and network management algorithm considered can cope with the dynamicity and disturbance in the network or not.

### 8.2. Evaluating Other Wireless Protocols or WirelessHART Itself

Protocol designers can use this implementation as a tool to compare the performance of other wireless protocols with WirelessHART. They can easily extend this simulator to develop new protocols or simulate other existing protocols, like the ISA100.11a standard. That way, they can assess whether a distributed management approach is better in coping with highly dynamic situations in a timely manner than centralized approaches, like WirelessHART, as we did in [32].

Designers can also test other network management algorithms and compare their performances. One can also modify the WirelessHART stack implementation and test various mechanisms used in different parts of the stack. For instance, the blind channel hopping and global blacklisting techniques, used in WirelessHART to mitigate external interferences and multi-path fading, can be substituted with other techniques, e.g., local blacklisting, and the performance of those schemes can be measured.

## 9. Conclusion and Future Work

We presented the complete implementation of the WirelessHART standard in the NS-2 network simulator and showed how this implementation can be used as a reference point to evaluate other wireless protocols, as well as to improve the WirelessHART stack and network management algorithm. Using sniffed traffic from the real WirelessHART network, we validated (i) the WirelessHART protocol stack and (ii) the network manager. Validation of the wireless protocol stack by using the captured traffic from other real WirelessHART networks is feasible, since our implementation effort follows the standard. However, since the standard does not specify the specific management algorithm, different implementation efforts have different characteristics. By comparing the real and simulated network managers, we found that differing network management algorithms might affect the performance of the WirelessHART network. However, it is expected and observed that the main requirements of the WirelessHART protocol, namely the provision of reliable and also real-time communication, should be fulfilled by different network manager algorithms. Consequently, when applying other network management algorithms, results may differ. Empirical analysis showed that the simulated results are quite close to the results obtained from real networks. Hence, we can make very realistic simulations with our implementation. We also demonstrate the versatility and usability of our implementation by showing some further evaluation results in diverse scenarios. In addition, the simulation model and scripts can be used for research purposes and will become available as an open source implementation in the near future.

## Figures and Tables

**Table 1.** Different classes of applications, as defined by the International Society of Automation (ISA).

**Category**	**Class**	**Application**	**Description**
Safety	0	Emergency action	Always critical

Control	1	Closed-loop regulatory control	Often critical
	2	Closed-loop supervisory control	Usually noncritical
	3	Open-loop control	Human in loop

Monitoring	4	Alerting	Short-term operational consequence
	5	Logging and downloading/uploading	No immediate operational consequence

**Table 2.** NS-2 simulation parameters.

**Parameter**	**Value**
Number of routers	Gateway, two access points
Number of I/O devices	4
Simulation area	100 × 100
Minimum superframe size (Real network)	128 slot
Minimum superframe size (Simulated network)	200 slot
Data rate	250 kb/s
Frequency band and channel	2.4 GHz, 11–26 channels
Radio range	≈40 m
Radio propagation model	Shadowing model
Path loss exponent	2.0
Shadowing deviation (dBm)	5.7 (Engineering building) and 8 (corridor)
Reference distance	1.0 m
Mac retransmission	3
Application traffic model	Constant Bit Rate (CBR)

**Table 3.** Periodic messages rates. CBR: constant bit rate.

**Item**	**Parameter**	**Value**	**Transmission type**
Simulated periodic management data	Neighbor health list	30 s	Acknowledged unicast
	Neighbor signal Level reports	30 s	Acknowledged unicast
	Advertisement rate	4 s	Unacknowledged broadcast

Real network periodic reports and advertisement	Advertisement rate	1.28 s	Unacknowledged broadcast
	Device health report	914 s	Acknowledged unicast
	Neighbor health list report	914 s	Acknowledged unicast
	Neighbor signal level report	914 s	Acknowledged unicast

Application data for real and simulated network	Sensor data rate	4 s and	Acknowledged unicast
		60 s	

**Table 4.** Energy-consumption parameters.

**Parameter**	**Value**	**Parameter**	**Value**
Radio chip	Dust [31]	Supply Voltage	3.76 V
Transmit power (0 dBm)	20.303 mW	Receive power	16.92 mW
Listen power	16.92 mW	Receive a packet	4.5 mA
Transmit at 0 dBm	5.4 mA	TsRxWait	2.2 ms
TsAck [1] (26 bytes)	0.832 ms	TsCCA [1]	0.128 ms
TsRxTx [1] (TxRx turnaround)	0.192 ms	TsMax [1] Packet (133 bytes)	4.256 ms

**Table 5.** Energy-consumption per transaction.

**Notation**	**Formula**	**Value**
Acknowledged Tx	TsCCA × Listen power + TsMaxPacket × Transmit power + TsAck × Receive power	102.6 *μJ*
Acknowledged Rx	TsMaxPacket × Receive power + TsAck × Transmit power	88.90 *μJ*
Broadcast Tx	TsCCA × Listen power + TsMaxPacket × Transmit power	88.57 *μJ*
Broadcast Rx	TsMaxPacket × Receive power	72.01 *μJ*
Idle	TsRxWait × Listen power	37.22 *μJ*

**Table 6.** Energy-consumption in the network (in 25,000 s) during normal operation. GW: gateway; NM: network manager.

**Scenario**	**Item**	**GW(NM)**	**Node 2**	**Node 3**	**Node 4**	**Node 5**
Real (Dust)	Total Energy (without idle listening)	9.80 J	1.93 J	1.98 J	1.96 J	0.64 J
	Total Energy (without Advand idle)	0.58 J	0.22 J	0.27 J	0.34 J	0.18 J

Simulation (Dust)	Total Energy (without idle listening)	7.60 J	1.56 J	2.74 J	2.85 J	1.64 J
	Total Energy (without Adv and idle)	1.47 J	0.19 J	0.72 J	1.15 J	0.60 J
